# Impact of telemonitoring systems in cancer care: a systematic review

**DOI:** 10.1136/bmjcsurg-2026-000038

**Published:** 2026-07-09

**Authors:** Theologia Tsitsi, Andreas Charalambous, Maria-Dolores Christofi, Constantina Cloconi, Koralia Michail, Victoria Leclercq, Efthyvoulos Kyriacou

**Affiliations:** 1 Nursing, Cyprus University of Technology, Limassol, Cyprus; 2 Cyprus University of Technology, Limassol, Cyprus; 3 Department of Nursing, University of Turku, Turku, Finland; 4 Department of Nursing, University of Maribor, Maribor, Slovenia; 5 Sciensano Cancer Centre, Brussels, Brussels, Belgium; 6 Electrical Engineering Computer Engineering and Informatics, Cyprus University of Technology, Lemesos, Cyprus

**Keywords:** Telemedicine in Surgery, Evidence-Based Surgery, Health Information Technology, Neoplasms, Surgical Procedures

## Abstract

This systematic review evaluates the impact, usability, technical performance and adherence of telemonitoring systems in adult cancer care. This systematic review followed Preferred Reporting Items for Systematic Reviews and Meta-Analyses and Peer Review of Electronic Search Strategies guideline. PubMed, MEDLINE and CINAHL were searched from June 2013 to March 2025. Search terms covered cancer care, telemonitoring systems, mobile health (mHealth), eHealth, health outcomes and evaluation tools. Included studies were randomised controlled trials, controlled clinical trials or prospective trials involving adult patients with cancer using telemonitoring systems for symptom management. Exclusion criteria included cross-sectional, retrospective or non-English studies. Two reviewers screened and extracted data, with a third reviewer resolving disagreements. Risk of bias was assessed using Cochrane Risk of Bias Tool V.1. Due to heterogeneity, a narrative synthesis was conducted, categorising studies by intervention type, clinical outcomes, adherence and usability. 16 studies were included. Telemonitoring appears to hold potential in oncology care, with indications of improved quality of life, symptom management and patient engagement, particularly when real-time clinician feedback is included. While mHealth apps were well received, asynchronous systems were associated with lower adherence. Few studies addressed data security or long-term outcomes. The search was aligned with European Union (EU) - assigned eHealth for Cancer Prevention & Care (eCAN Joint Action) priorities, which may have narrowed the results and limited the overall completeness of this systematic review. Telemonitoring shows promise in oncology care but requires personalisation, workflow integration and long-term evaluation. Real-time clinician support may enhance adherence and outcomes.

PROSPERO registration number

CRD42023430639.

**WHAT IS ALREADY KNOWN ON THIS TOPIC**

- Telemonitoring platforms in oncology have shown promise for symptom tracking and quality-of-life improvement, but adherence remains variable and long-term effectiveness is poorly established.

**WHAT THIS STUDY ADDS**

- Real-time clinician feedback was consistently associated with better adherence and clinical outcomes compared with asynchronous, self-guided systems, while few studies addressed long-term outcomes or data security.

**HOW THIS STUDY MIGHT AFFECT RESEARCH, PRACTICE OR POLICY**

- Future telemonitoring systems should prioritise real-time clinician support and standardised outcome measures, and whether telemonitoring can be integrated into routine postoperative surgical oncology care pathways remains an important area for future research.

## Introduction

 Patients with cancer monitor symptoms at home using wearable devices or mobile apps, but delays in symptom detection can lead to preventable hospitalisations. Telehealth, including telemedicine, telemonitoring and mobile health (mHealth), offers remote support through virtual consultations and real-time symptom tracking. mHealth tools and patient-reported outcome (PRO) measures aid in symptom management, medication adherence and clinical communication. Although these technologies show promise in improving oncology care and quality of life (QoL), evidence regarding their clinical and cost-effectiveness is still emerging, and integration into standard clinical workflows remains limited. For the purposes of this review, ‘telemonitoring systems’ refer to integrated digital platforms or technologies, such as mobile apps, wearable sensors and web-based dashboards, that enable continuous remote monitoring and communication between patients with cancer and their care teams.

As telemedicine becomes more prevalent, there are challenges apparent. Therefore, the European Commission has set up a joint action strengthening eHealth, including telemedicine and remote monitoring for healthcare systems for cancer prevention and care, called eCAN.^1^ It examines how telemedicine and remote monitoring work in practice, how effective they are and how they can be better used to reduce inequalities in cancer care within the EU.

Based on the actions of the eCAN Joint Action project, this systematic review expands on previous reviews, such as those by Warrington *et al*^2^ and da Silva *et al*,^3^ by providing a more comprehensive evaluation of telemonitoring systems in cancer care. While Warrington *et al*^2^ primarily focused on electronic symptom reporting, the present review expands to include real-time monitoring through telemonitoring systems delivered via mHealth platforms. While da Silva *et al*^3^ examined the feasibility of telehealth for patients with head and neck cancer, this review assesses telemonitoring interventions across various cancer types. Additionally, unlike previous reviews, this systematic review analyses adherence rates.

Another review on telehealth engagement in patients with advanced cancer^4^ examined patient interaction with telehealth interventions but did not extract data on technical robustness or security aspects. Similarly, a recent meta-analysis by Wang *et al.*^5^ examined Remote Patient Monitoring Software (RPMS) interventions, focusing on their impact on PROs, QoL, symptom burden and self-efficacy. Their findings suggest that RPMS interventions can enhance symptom management and improve patient engagement in oncology care. However, their review primarily assessed mobile and web-based telemonitoring systems designed to facilitate patient self-reporting and health information provision, with less emphasis on adherence tracking.

In addition, a recent scoping review by Amarelo *et al*^6^ explored the use of technological tools to support physical rehabilitation in patients with cancer undergoing chemotherapy. While their work provides a broad overview of digital interventions in supportive care, it does not evaluate real-time monitoring systems, adherence rates or clinical outcomes, which are central to the present review.

This systematic review aims to evaluate the impact of telemonitoring systems in cancer care by examining their clinical outcomes, technical features, patient acceptability and adherence. Methodologically, this systematic review applies a more rigorous approach by including only randomised controlled trials (RCTs) and prospective clinical trials, ensuring higher-quality evidence compared with past reviews that included feasibility studies, cross-sectional research and qualitative studies.

## Methods

### Design

This systematic review followed the guidelines provided by the Preferred Reporting Items for Systematic Review and Meta-Analysis.^7^ The review was registered on PROSPERO (ID): CRD42023430639.

### Deviations from protocol

Several modifications were made to the original protocol. Although the initial scope was limited to studies from EU countries, this was broadened internationally due to a limited number of eligible studies and to improve the applicability of findings.^8^ The criterion requiring patients to have documented access to telemedicine was also removed, as many studies did not explicitly state this, and excluding them risked omitting data.^9^ Lastly, the focus shifted from satisfaction and efficiency to pain and symptom management, aligning with WHO priorities in cancer care^10^ and existing evidence of telemedicine’s role in addressing these clinical needs.^11^

### Patient and public involvement

None.

### Search strategy

The search strategy was developed to meet the goals of the eCAN Joint Action project and aligned with its priorities and was peer-reviewed in accordance with the Peer Review of Electronic Search Strategies 2015 guideline.^12^ The thematic blocks were designed to reflect the project’s clearly defined areas of interest. The full search strategies for all databases, including all filters, limits and search terms used, are provided in online supplemental file S1.

### Inclusion/exclusion criteria

Inclusion criteria were defined using the Population, Intervention, Comparison, and Outcomes (PICO) framework. Eligible studies included patients aged 18 years or older undergoing cancer treatment for a new diagnosis or recurrence. The intervention involved telemedicine tools such as video consultations, virtual follow-ups, or remote monitoring tools (e.g., wearable devices or mobile apps for symptom reporting). Studies had to include a comparator, such as standard cancer care or no intervention, and focus on outcomes related to pain alleviation or cancer symptom management. Only English-language studies with RCTs, cluster RCTs, controlled clinical trials, or prospective clinical trials were included. Exclusion criteria comprised studies with patients under 18 years old or those using cross-sectional, case study, case–control, or retrospective comparative cohort designs or review formats.

### Study selection

**Figure 1:**
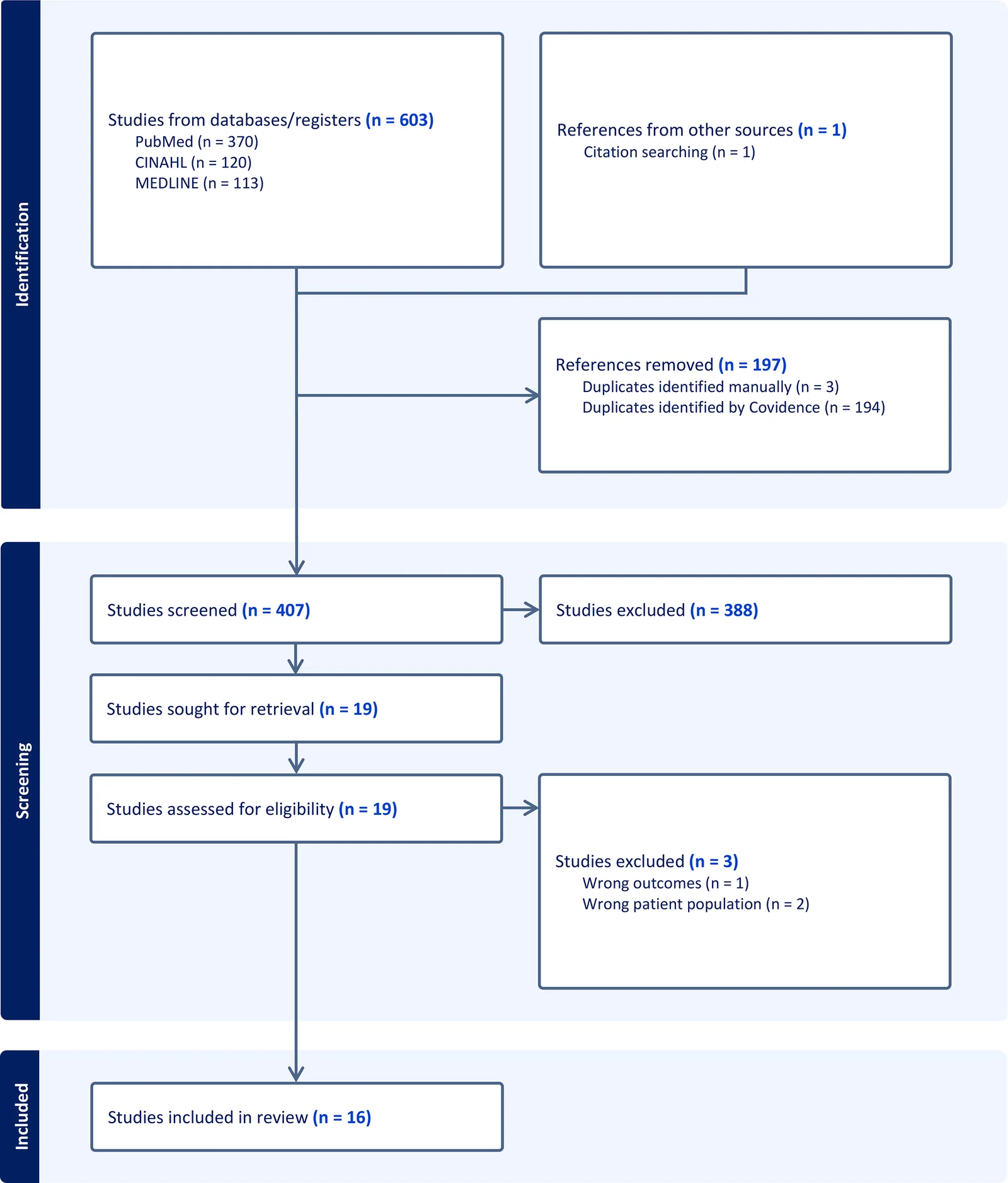
Preferred Reporting Items for Systematic Reviews and Meta-Analyses flowchart of the identification and screening process.

All articles were exported to ‘Covidence’,^13^ a platform to facilitate study selection and data management. Two independent reviewers (TT and M-DC) screened the titles and abstracts of the retrieved studies against the predefined eligibility criteria. A third reviewer (KM) acted as a moderator in cases of disagreement. After removing duplicates and irrelevant articles, remaining full texts were assessed using inclusion and exclusion criteria. In total, 16 studies met the eligibility criteria (figure 1).

### Data extraction

Due to the heterogeneity of interventions, a formal meta-analysis was not feasible. Instead, a narrative synthesis approach was applied, categorising studies based on telemonitoring methods, primary outcomes, clinical impact/effectiveness, patient adherence and usability/acceptance.

To ensure systematic reporting, extracted data included (1) study, (2) country, (3) purpose, (4) description of the intervention, (5) population—number of participants allocated to the intervention and control group, (6) outcomes, and (7) results (Table 1).

**Table 1: Characteristics:** of studies included in the systematic review (intervention, setting, sample size, outcomes and key findings)

Study/country/purpose	Intervention	Population	Outcomes	Results (mean/SD, p value)/adherence/engagement findings
Baik *et al*, USA Test web-based supportive care in adult cancer.^25^	PtCareAnywhere web-based supportive care system (12-week programme).	Adults with breast, lung or PC; n=72 (I=36; C=36)	FACT-G, SEMCD	Intervention users had a 1.53-point greater reduction in SDS-15 scores (95% CI 0.32 to 2.75; p=0.01). Adherence: ≥2 ESRA-C exposures defined use; 62% met this criterion, with ↑ use among radiation oncology and more educated/working pts.
Berry *et al*, USA Assess web symptom/QoL reporting on distress.^18^	ESRA-C web symptom/QoL self-report tool during therapy versus usual care.	Pts starting cancer therapy (n=752) I: 374, C: 378	SDS-15	Greater reduction in SDS-15 scores in the IC (Δ −1.53; 95% CI 0.32 to 2.75; p=0.01). 62% had ≥2 ESRA-C exposures and 34% ≥1 voluntary session; ↑ use in radiation oncology and among more educated/working pts.
Hauffman *et al*, Sweden Reduce anxiety and depression in pts with cancer.^29^	iCAN-DO stepped-care portal (psychoeducation+guided iCBT) versus standard care.	Pts with cancer (n=245) I: 124, C: 121	HADS-A/D, STAI-S, MADRS-S, EROS, EORTC QLQ-C30, FACIT-F, ISI, PCL-C	Compared with standard care, iCAN-DO led to a small but statistically significant ↓ in depression (HADS-D; adjusted mean difference −0.54; 95% CI −1.08 to −0.01; p<0.05), with no significant between-group effects on anxiety, post-traumatic stress or HRQoL. A clinically meaningful >20% ↓ in depression over 10 months was observed only in the iCAN-DO group.
Hoek, the Netherlands Assess telepalliative consultations on symptom burden.^30^	Weekly video teleconsultations with specialist palliative care team versus standard care.	Pts with advanced cancer at home (n=74) I: 38 C: 36	ESAS, TDS HADS	TDS was significantly higher in the IG at week 12 versus CG (adjusted difference 6.90; 95% CI 0.17 to 13.63; p=0.04). HADS-A scores were also higher in the IG (estimate 1.40; 95% CI 0.14 to 2.66; p=0.03), with no difference in HADS-D (estimate 0.30; 95% CI −1.39 to 1.99; p=0.73). No significant differences in unmet needs, continuity of care, hospital admissions or caregiver burden. Adherence was low: 42% completed 12-week follow-up, with high attrition in both groups due to deterioration or death; completion rate of teleconsultations was not reported.
Hou *et al*, Taiwan Support self-management after first BC diagnosis.^20^	BCSMS mHealth self-management app for 12 weeks versus usual care.	BC (stages 0–III) (n=112) I: 53 C: 59	QoL (EORTC QLQ-C30, QLQ-BR23)	At 3 months, QoL was higher in the IG versus CG (QLQ-C30 summary: 83.45 vs 82.23; QLQ-BR23: 65.53 vs 63.13). GEE showed significantly greater improvement with the app (QLQ-C30 ΔT2–T0: 8.98 vs 3.93; between-group difference 5.05; p=0.03; QLQ-BR23 ΔT2–T0: 5.85 vs 1.92; difference 3.93; p=0.04). Follow-up completion was high (89.3% at 3 months), but actual app use frequency was not recorded.
Huggins *et al*, Australia Compare nutrition counselling: telephone versus app versus usual care.^16^	Dietitian-led nutrition counselling via telephone or app versus usual care (3-arm RCT).	Pts with upper GI cancer; n=111 (control=37; telephone=38; app=36).	EQ-5D-5L (QALYs), EORTC QLQ-C30, PG-SGA	No sig. between-group differences in QALYs, QoL (EORTC QLQ-C30) or nutritional status (PG-SGA); 12-month survival was similar across arms. The asynchronous app group showed more withdrawals and lower acceptability than the telephone counselling group.
Ji *et al*, South Korea Evaluate mHealth home pulmonary rehabilitation for NSCLC.^21^	efil breath mHealth home pulmonary rehab (fixed vs fixed interactive).	Pts with NSCLC; n=64 (fixed=32; fixed-interactive=32).	6MWD, mMRC, EQ-VAS, EQ-5D.	Across groups, 6MWD improved from 433.43 to 471.25 m (p=0.001); mMRC from 0.94 to 0.61 (p=0.02); EQ-VAS from 76.05 to 82.09 (p=0.002); EQ-5D changes were not significant. No significant differences were found between fixed and fixed-interactive regimens. Compliance and progress were monitored via the app and website; 43/64 participants (~67%) completed the 12-week evaluation, but no formal adherence percentage was reported.
Lally, USA Reduce early postdiagnosis distress in BC.^26^	CaringGuidance unguided web-based psychoeducational/CBT programme versus usual care.	Women with stage 0–IIBC≤3 months post diagnosis; n=100 (I=57; C=43).	DT, CES-D, IES; Brief COPE (moderator)	No sig. overall time×group interaction for DT, CES-D or IES. Post hoc analyses showed greater reductions in distress (DT; p=0.03) and depressive symptoms (CES-D; p=0.043) between months 2 and 3 in the IG versus CG, with no significant effect on IES (p=0.18). Effects were stronger among women with higher baseline distress and lower baseline active coping. Among 54 women with complete usage data, median use was ~5 hours (~15 sessions) over 12 weeks, with wide variability (0–26 sessions).
LeBlanc *et al*, USA Test symptom self-management app feasibility and impact.^22^	Blood Cancer Coach symptom self-management app versus usual care.	MM and CLL pts n=72 (I: 33; C: 39)	PROMIS Global Health (GPH, GMH), PCL-5, ESAS-r, perceived helpfulness and satisfaction	No significant between-group differences at week 4 in GPH (p=0.84), GMH (p=0.94), symptoms (p=0.21) or PTSD (p=0.50). Intervention participants completed on average ~148 app tasks over 8 weeks; most-used features were the medication log and distress tracking. Among those completing the week-8 satisfaction survey (39% response), 73% reported at least moderate overall satisfaction with the app; no formal adherence threshold was predefined.
Merz, USA Test supportive-care app feasibility and usability.^17^	D-SCAN supportive-care and symptom-tracking app versus usual care.	Pts with advanced cancer/caregivers; 50 pts (I=25; C=25) + 10 caregivers (intervention).	FACT-G, CarGOQOL, ESAS-r, PAM-13, feasibility, NPS, resource awareness/use	Small improvements in QoL and activation (FACT-G+1.1; CarGOQOL+2.2; p=0.41; PAM-13+1.6). Pt NPS was 14.3% and caregiver NPS 12.5%. Resource awareness ↑ (+3.7 for pts; +4.1 for caregivers) with slight ↑ in utilisation (+0.8 and +0.6 items, respectively). Feasibility target was met: 76% of IG pts fulfilled predefined criteria (≥3/12 ESAS-r surveys, exit survey completion, phone returned), exceeding the 75% feasibility threshold.
Park *et al*, South Korea Evaluate AR-based home telerehabilitation after breast surgery.^19^	UINCARE Home+AR telerehabilitation versus brochure-based home exercises.	Pts with postoperative BC with limited shoulder ROM; n=100 (I=50; C=50).	FACT-B, active/passive shoulder ROM, NRS pain, QuickDASH, EQ-5D-5L.	Both groups showed significant improvements in shoulder ROM, pain, function and QoL from baseline to 12 weeks (all p<0.001), with no sig. between-group differences. Exercise adherence over 8 weeks was lower in the UINCARE group (36.1±14.5 days) than in the CG (45.1±13.1 days; p=0.002).
Sprave *et al*, Germany Assess daily ePRO app during radiotherapy.^24^	Daily ePRO (myoncare) app during radiotherapy versus standard monitoring.	Head and neck cancer n=100 randomised (I: 50; C: 50; analysed: I: 45; C: 48).	EORTC QLQ-C30, QLQ-H&N35, PSQ-18.	EORTC QLQ-C30 global health/most domains were similar between groups, but financial difficulties were ↑ in the ePRO arm (p=0.005). QLQ-H&N35 showed ↑ symptom burden with ePRO across multiple symptoms (eg, pain, swallowing, dry mouth/sticky saliva, coughing/speech, social eating/contacts, feeling ill, supplements/feeding tube, weight gain; all p≤0.03; large effects). PSQ-18 showed higher pt satisfaction in 3/7 domains (interpersonal manner, financial aspects, time with doctor). Adherence was excellent: 100% of ePRO pts answered ≥80% of daily questions, and >80% answered ≥ 80% of weekly items.
Sun *et al*, China Evaluate MTM-based mHealth intervention in DTC.^15^	MTM-based mHealth programme via WeChat plus usual care versus usual care alone.	Pts with DTC post-surgery, pre-I-131/TST; n=104 analysed (I=62; C=42; 111 randomised).	PHQ-4 FCR-4, EQ-5D-5L CSQ-3	The IC showed a significant ↓ in PHQ-4 total scores, whereas controls showed no change (p<0.026); anxiety and depression subscale scores were significantly ↓ in the IC at follow-up (p<0.015 and p<0.032). Fear of cancer progression ↑ in the CG but not in the IC (between-group p<0.001). After 3 months, EQ-5D-5L anxiety/depression and VAS scores were better in the IC than in controls (p≈0.03), with no meaningful change in controls. Adherence was high: 104/111 pts (93.7%) completed the 3-month intervention (6.3% lost to follow-up).
Wennerberg *et al*, Sweden Support self-care after radical prostatectomy.^27^	ePATH web/mobile self-care platform for 12 months post-RP versus standard care.	Men postradical prostatectomy; n=170 (I=85; C=85).	EPIC, Saltin-Grimby Physical Activity Level Scale	No sig. intervention effects on continence function (β = −5.60; p=0.09; 95% CI −12.15 to 0.96) or sexual function (β = −0.12; p=0.97; 95% CI −7.05 to 6.81).
Wittmann *et al*, USA Support sexual recovery in couples with PC.^28^	TrueNTH Sexual Recovery interactive web programme versus standard online information.	Couples facing treatment for localised PC; n=142 couples (I=62; C=80).	PROMIS GSSL; PROMIS Sexual Interest/Activity/Aids; EPIC-26 sexual; FSFI; IIEF	No sig. group differences in GSSL scores 6 months post-treatment (p=0.4). At 3 months post-treatment, intervention couples reported more engagement in penetrative and non-penetrative sexual activities than controls (p=0.05). Participant satisfaction was high: >73% reported high or moderate satisfaction with the module content, and >85% would recommend the intervention to other couples.
Wolff *et al*, Germany Test lifestyle-coaching app on fatigue and distress.^23^	PINK! lifestyle-coaching app for 12 weeks versus waiting-list control.	Pts with BC/survivors in therapy or aftercare; n=60 (I=38; C=22).	PHQ-9, EORTC QLQ-C30 fatigue, IPAQ (MET-min)	In the IC, PHQ-9 scores ↓ by 2.5 points (~32.9% reduction; p<0.01; d=0.8) and fatigue scores ↓ by 9.9 points (~19.5% reduction; p<0.01), with ↑ physical activity, particularly among those with high baseline fatigue. A dose–response pattern was observed across app-usage groups (>200, 100–200, <100 min/12 weeks), with the highest-use group showing the greatest reductions in distress and fatigue; overall dropout was 11.7%.

AR, augmented reality; BC, breast cancer; BCSMS, Breast Cancer Self-Management Support; CarGOQOL, Caregiver Oncology Quality of Life questionnaire; CES-D, Center for Epidemiologic Studies Depression Scale; CG, control group; CLL, Chronic Lymphocytic Leukemia; CSQ-3, Client Satisfaction Questionnaire-3 (item version); DT, Distress Thermometer; DTC, differentiated thyroid cancer; EORTC QLQ-C30, European Organisation for Research and Treatment of Cancer Quality of Life Questionnaire-Core 30; ePATH, electronic Patient Activation in Treatment at Home; EPIC, Expanded Prostate Cancer Index Composite; ePRO, electronic pt-reported outcome; EQ-5D, EuroQol 5-Dimension questionnaire; EQ-5D-5L, EuroQol 5-Dimension 5-Level questionnaire; EQ-VAS, EuroQol Visual Analogue Scale; EROS, Environmental Reward Observation Scale; ESAS-r, Edmonton Symptom Assessment System Revised; ESRA-C, Electronic Self-Report Assessment for Cancer; FACIT-F, Functional Assessment of Chronic Illness Therapy-Fatigue; FACT-B, Functional Assessment of Cancer Therapy-Breast; FACT-G, Functional Assessment of Cancer Therapy-General; FCR-4, Fear of Cancer Recurrence-4 (item scale); FSFI, Female Sexual Function Index; GEE, Generalized Estimating Equations; GMH, Global Mental Health; GPH, Global Physical Health; GSSL, Global Satisfaction with Sex Life (PROMIS scale); HADS, Hospital Anxiety and Depression Scale; HADS-A, Hospital Anxiety and Depression Scale-Anxiety; HADS-D, Hospital Anxiety and Depression Scale-Depression; HRQoL, health-related quality of life; IC, intervention group; iCAN-DO, Name of the internet-based stepped-care intervention evaluated in the AdultCan trial; iCBT, internet-based Cognitive Behavioral Therapy; IES, Impact of Event Scale; IG, intervention group; IIEF, International Index of Erectile Function; IPAQ, International Physical Activity Questionnaire; ISI, Insomnia Severity Index; MADRS-S, Montgomery-Åsberg Depression Rating Scale-Self-report; MET, Metabolic Equivalent of Task; mHealth, mobile health; MM, Multiple Myeloma; mMRC, Modified Medical Research Council; MTM, Multi-Theoretical Model of health behavior change; 6MWD, Six-Minute Walk Distance; NPS, Net Promoter Score; NRS, Numerical Rating Scale; NSCLC, non-small cell lung cancer; PAM-13, Patient Activation Measure; PC, prostate cancer; PCL-5, PTSD Checklist for Diagnostic and Statistical Manual of Mental Disorders, Fifth Edition (DSM-5) ; PCL-C, Post-Traumatic Stress Disorder (PTSD) Checklist-Civilian Version; PG-SGA, Patient-Generated Subjective Global Assessment; PHQ, Patient Health Questionnaire; PROMIS, Patient-Reported Outcomes Measurement Information System; PSQ-18, Patient Satisfaction Questionnaire-18 (short form); pts, patients; QALY, quality-adjusted life year; QLQ-BR23, EORTC Quality of Life Questionnaire-Breast Cancer Module (23 items); QLQ-H&N35, EORTC Quality of Life Questionnaire-Head and Neck Cancer Module (35 items); QoL, quality of life; QuickDASH, Quick Disabilities of the Arm, Shoulder and Hand questionnaire; RCT, randomised controlled trial; ROM, range of motion; RP, radical prostatectomy; SDS-15, Symptom Distress Scale (15-item); SEMCD, Self-Efficacy for Managing Chronic Disease; STAI-S, State-Trait Anxiety Inventory-State subscale; TDS, Total Distress Score; TST, Thyrotropin (TSH) Suppression Therapy; UINCARE, proprietary brand name of the AR-based telerehabilitation system (not an acronym).

## Results

### Rate of agreement

Interrater reliability was assessed using Cohen’s kappa (κ), with a κ value above 0.75 indicating strong agreement.^14^ During the title and abstract screening phase, the reviewers achieved a proportionate agreement of 85.98%, with a Cohen’s kappa of 0.33. In the full-text review phase, the agreement improved substantially, with a proportionate agreement of 94.12% and a Cohen’s kappa of 0.77, indicating substantial agreement between reviewers (Table 2). The full contingency table and probability calculations are provided in online supplemental file S2. Any discrepancies in study inclusion were discussed among reviewers (TT and M-DC) with a third reviewer (KM) moderating.

**Table 2:** Interrater reliability per screening phase

			Cohen’s kappa
Screening phase	Reviewer pair	Proportionate agreement	
Title/abstract	TT and M-DC	0.85979 (85.98%)	0.32911 (fair)
Full-text review	TT and M-DC	0.94118 (94.12%)	0.76712 (substantial)

### Risk-of-bias assessment

Table 3 summarises the risk of bias assessment for the 16 studies included in this review, evaluated using the Cochrane Risk of Bias Tool V.1 across seven domains: sequence generation, allocation concealment, blinding of participants and personnel, blinding of outcome assessors, incomplete outcome data, selective reporting and other sources of bias. Each domain was rated as low, high or of some concern. Two investigators (TT and M-DC) independently assessed study quality and compared ratings. A third investigator (KM) resolved disagreements.

**Table 3: Risk-of-bias:** assessment for included studies

Study ID	Sequence generation	Allocation concealment	Blinding of participants and personnel for all outcomes	Blinding of outcome assessors for all outcomes	Incomplete outcome data for all outcomes	Selective outcome reporting	Other sources of bias
Baik *et al*^25^	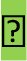	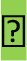	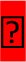	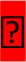	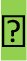	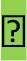	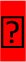
Berry *et al*^18^	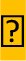	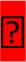	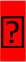	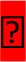	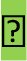	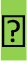	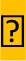
Hauffman *et al*^29^	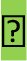	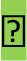	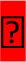	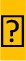	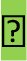	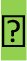	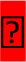
Hoek *et al*^30^	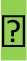	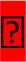	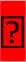	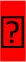	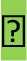	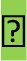	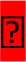
Hou *et al*^20^	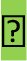	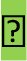	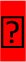	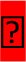	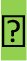	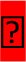	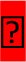
Huggins *et al*^16^	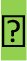	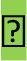	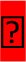	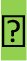	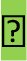	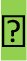	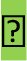
Ji *et al*^21^	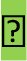	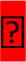	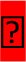	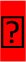	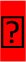	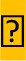	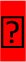
Lally *et al*^26^	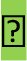	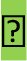	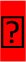	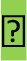	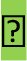	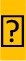	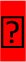
LeBlanc *et al*^22^	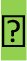	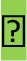	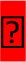	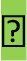	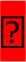	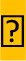	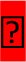
Merz^17^	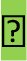	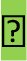	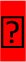	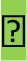	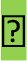	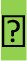	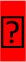
Park *et al*^19^	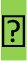	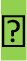	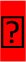	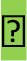	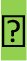	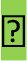	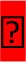
Sprave *et al*^24^	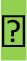	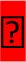	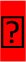	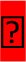	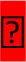	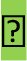	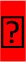
Sun *et al*^15^	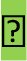	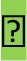	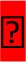	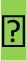	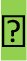	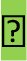	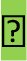
Wennerberg *et al*^27^	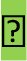	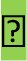	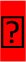	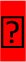	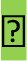	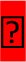	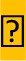
Wittmann *et al*^28^	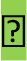	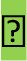	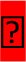	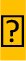	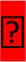	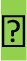	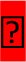
Wolff *et al*^23^	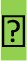	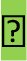	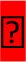	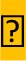	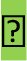	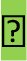	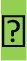

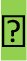
 indicates low risk of bias, 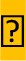 indicates unclear risk of bias, 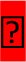 indicates high risk of bias.

All studies were judged to have a high risk of bias for blinding of participants and personnel, which is expected given the nature of digital and behavioural interventions where blinding is often not feasible. Only two studies^15 16^ were rated as low risk in six of seven domains. 14 studies demonstrated low risk for sequence generation. Overall, while the studies were methodologically sound in key areas, the inability to fully blind participants and staff may have introduced bias in outcome reporting or patient-reported data.

### Study selection and characteristics

The initial search across all databases yielded 604 records: 370 from PubMed, 120 from CINAHL, 113 from MEDLINE Complete and one reference from other sources. After removing duplicates and screening for eligibility, 16 studies were included in the systematic review (figure 1). The studies consisted of 10 RCTs and 3 pilot/feasibility RCTs and 3 prospective clinical trials. The total sample size across studies was 2345 individual participants, including 10 caregivers, with study sizes ranging from 50 participants^17^ to 752 participants.^18^

### Types of telemonitoring systems used

Interventions fell broadly into three categories: (1) mobile app-based systems, such as multitheoretical mHealth and symptom self-management apps^15^,^1719^; (2) web-based platforms, including supportive care portals and psychoeducational programmes^1825^,^29^; and (3) teleconsultation-based interventions delivered by telephone or video.^16 30^ Some systems combined web and mobile components.^27^ These platforms supported symptom reporting, medication tracking, psychological support and self-management.

### Duration of interventions

Intervention duration most commonly ranged from 8 to 12 weeks for mHealth and eHealth programmes,^1517 19^,^24 26^ while several web-based or teleconsultation interventions extended up to 6–12 months.^1627^,^29^ Follow-up assessments at 3, 6 or 12 months were included in some studies,^16 27 28^ allowing limited evaluation of medium-term effects.

### Technical evaluation of telemonitoring systems

The included studies assessed both technical and clinical aspects to evaluate system usability, feasibility, functionality and data protection. Some trials used standardised usability instruments such as the System Usability Scale,^25^ while others relied on individualised satisfaction or acceptability questionnaires.^21 22 24^ Feasibility was commonly examined using app interaction data, adherence logs and completion rates of electronic PROs (ePROs).^22 24 27^ Some trials reported technical issues and described real-time data transfer to central databases.^21 22 24^ In terms of security, platforms reported secure log-in or password-protected access, pseudonymised or deidentified data handling and encrypted data storage or transmission, often governed by institutional or national data protection oversight.^22 24 25 27^ These characteristics are summarised in table 4, which outlines the technical structure of each telemonitoring system.

**Table 4: Technical:** characteristics of telemonitoring systems

Study	Technology/intervention	Key technical characteristics
Baik *et al*^25^	PatientCareAnywhere web-based supportive care system (12-week/3-month programme)	Automated alerts: automatic alert messages for abnormal screening results sent to the clinical team. Personalised self-management resources: individualised education content based on screening/assessment results. Secure communication/sharing controls: communication platform for carers/family/friends to interact with the patient; patients control who is in their ‘circle of care’ and how much is shared. Social support integration: social-support functions to engage carers/family/friends/community resources (and community support resources/referrals based on screening). Device compatibility and EHR integration: optimised display for different devices (eg, smartphones/tablets); EHR integration listed as a key feature.
Berry *et al*^18^	ESRA-C: self-report web tool for symptom and QoL tracking (6–8 weeks)	Participants could self-monitor symptoms and quality-of-life issues; provided tailored self-care instruction for SxQOL issues; and provided communication coaching on how to report concerns to clinicians. Access methods: web-based access from home via the Internet. In-clinic access via a touch-screen computer; participants without Internet access could use a study tablet in the clinic (with research coordinator support). Components: teaching tips and viewing my reports (including line-graph tracking over time; pushed tips after moderate-to-severe reports).
Hauffman *et al*^29^	iCAN-Do stepped care (psychoeducation + internet-based CBT via U-CARE portal)	Platform: delivered through the U-CARE portal (internet-based infrastructure for delivering/evaluating interventions and collecting self-reported data). Access: Participants received login details through automated e-mails. Comms: all contacts with participants occurred through written messages in the portal. Step 1 (psychoeducation/self-care): available from randomisation throughout the study period; includes a library with 16 modules (short psychoeducative lectures in audiovisual and text formats), a peer-support moderated discussion forum, and ‘Ask an Expert’ (questions to a nurse + anonymised Q&A in an FAQ). Step 2 (iCBT): for participants with remaining symptoms (HADS>7 on any subscale) After Step 1: guided 10-week iCBT alongside Step 1; includes psychoeducation, self-monitoring, assignments and weekly psychologist guidance via written messages in the portal; participants choose among 15 modules and can undergo iCBT once (at 1, 4 or 7 months post-randomisation).
Hoek *et al*^30^	Weekly video teleconsultations (videoconferencing) with palliative care team (12 week data collection; weekly sessions scheduled for 13 weeks)	Teleconsultation modality: real-time (synchronous) audio-visual videoconferencing (‘teleconsultations’). Frequency and scheduling: weekly, prescheduled teleconsultations; initiated at the agreed time by an SPCT member (mostly the nurse practitioner). Equipment/software (home installed): a teleconsultation device was installed at the patient’s home. Devices initially were a Pal4 ‘Bidibox’ desktop (touch screen+separate microphone/speaker+separate camera) running the Pal4 application; later replaced by tablet computers (iPad 2/iPad mini) using FaceTime. Communication pathways outside scheduled calls: participants could not directly contact SPCT members via teleconsultation between scheduled sessions; when needing medical advice, they were encouraged to contact their GP, and if necessary the SPCT could be reached by phone. Structured consultation content: a predefined consultation schedule was available to SPCT members to ensure all palliative care domains were covered. Care team/GP involvement: the patient’s GP was invited to join teleconsultations; if not possible, SPCT contacted the GP by phone after the first teleconsultation, and SPCT was encouraged to send letters to the GP after the first and last teleconsultation.
Hou *et al*^20^	BCSMS mHealth app (self-management for breast cancer) (12 weeks/3 months)	Platform: mobile app used on participants’ iOS/Android phones; downloadable for widespread use (search ‘ibreast’ on iOS; ‘pink passport’ on Android). Access/evaluation: baseline assessment used paper-based questionnaires; follow-up evaluations at 1.5 and 3 months used paper-based or web-based questionnaires (participant choice). Security: Participant data were anonymised and stored on an encrypted, password-protected server. Tech support: app installed with remote technology support; support provided for technical problems (e.g., log-in and data entry problems). Prompts: Participants could use the app any time as needed; no prompts/reminders from the study team.
Huggins *et al*^16^	Nutrition counselling via telephone or app (18 weeks)	Platform: telephone (synchronous) or internet-enabled mobile app ‘myPace’ (asynchronous). Goals/tracking: goals were set and goal achievement monitored; myPace enables participants to self-monitor goal attainment and body weight. Education/guidance: behavioural-based, individually tailored, symptom-directed nutrition intervention with tailored recommendations and guidance on how to perform the behaviour. Review schedule: reviews were planned weekly or fortnightly (depending on need).
Ji *et al*^21^	‘efil breath’ mHealth pulmonary rehabilitation platform (12 weeks)	UI: app screenshots include screens for the 6MWT and exercise routine (fixed exercise programme). Real-time data: the platform includes a patient monitoring website that collects data from the apps in real time; health data (eg, 6MWT results, rehab progress, heart rate, breathing difficulty) are sent from the apps to a database. Clinician access: lung cancer specialists and nurses accessed patient data through the monitoring website and were permitted to view only their own patients’ data; compliance and progress were monitored for timely intervention.
Lally *et al*^26^	CaringGuidance web programme (psychoeducation+CBT) (12 weeks)	Platform: web-based program; required email + email+internet on a desktop or laptop (not mobile capable at that time). Usage analytics: an embedded data analytics system monitored use (minutes, number of sessions, mean log-in duration, and type of material accessed). UI: user-guided, self-tailored navigation/flexibility to explore content. Modules: five modules/17 subtopics (e.g., managing thoughts about cancer/diagnosis, body image, receiving support, moving forward). Engagement: programme engagement encouraged through automatically generated emails.
LeBlanc *et al*^22^	Blood Cancer Coach: a fully automated mHealth app for MM/CLL symptom self-management (8 weeks)	Platform: mobile app via Pattern Health; participants needed a smartphone (iPhone or Android); access codes were used to download; the app also administered consent, randomisation and surveys. Data collection: patient-reported data collected through the app at baseline, 4 weeks and 8 weeks. Automation: fully automated; includes symptom/distress tracking via notifications and provides tailored feedback based on severity. Security: data encrypted in transit and at rest; locally stored data encrypted; study-team access password-protected and limited; analyses conducted on deidentified data.
Merz^17^	D-SCAN app for symptom tracking+supportive care resource awareness/navigation (12 weeks)	Platform: Participants were provided a secure study mobile phone for the study duration with access to only the D-SCAN app. Security: described as a secure study phone (restricted access to only the study app). Symptom tracking: the app prompted completion of ESAS-r symptom assessments at least weekly (12 weekly surveys). Tailored care-resource recommendations: based on ESAS-r responses, the app provided relevant supportive care service/content recommendations (linked to the cancer support programme's services). UI: not described.
Park *et al*^19^	UINCARE Home+AR telerehabilitation system with motion tracking (8-week daily exercise; follow-up to 12 weeks)	Real-time motion tracking and feedback during exercises (e.g., sound + visual cues on screen). Customised exercise programmes prescribed/implemented by physicians via the internet; physicians could adjust exercise difficulty based on performance. Remote monitoring: exercise performance parameters (e.g., repetitions, movement accuracy) recorded in real time on an internet server for physician review/feedback. Interactive exercise guidance: on-screen instructions; sensor calibration before exercise; participants could stop or skip an exercise. Home installation: system installed in patients’ homes so they could perform rehabilitation at home (hardware-assisted telerehabilitation).
Sprave *et al*^24^	Daily symptom reporting via ePRO app during radiotherapy (mean treatment time ~44 days)	The mobile app facilitated daily reporting of treatment-related symptoms and quality-of-life items immediately prior to each radiotherapy fraction (seven items/day; items rotated so all were answered weekly). Patients were asked daily via the app about their need for a physician appointment. The app (myoncare) was used on a departmental (department-owned) mobile device.
Sun *et al*^15^	MTM-based mHealth intervention delivered via WeChat (12 weeks/3 months)	Platform: implemented on an ‘intelligent’ WeChat platform (participants used smartphones and were familiar with WeChat). Communication: supports direct doctor–patient communication for timely professional answers. Reminders/real-time support: clinicians could provide real-time interventions via WeChat, including medication-time reminders and thyroid-cancer knowledge dissemination. Educational content, delivered through WeChat group health education, online health lectures and personalised health education programmes, continued for 3 months after discharge. Monitoring/support: included drug supervision as part of comprehensive care.
Wennerberg *et al*^27^	ePATH web/mobile for self-care support at home (12 months post-radical prostatectomy)	Platform: web and mobile app. Security: Participants needed a facility for secure mobile log-in; a secure pathway was used for messaging with cancer nurse specialists. UI/individualisation: Nurse-assisted, individualised, interactive self-care modules tailored to patient needs; ePATH was tested for quality/usability with end-user groups. reminders via app notifications, graphs, secure messaging with daily nurse responses, and log data from the server.
Wittmann *et al*^28^	TrueNTH sexual recovery web programme (6 modules over 7 months)	Platform: tailored, interactive, web-based online tool. Tailoring: tailored to treatment type (surgery/radiation) and sexual orientation (female/male partner). Content/delivery: 6 modules accessed over a 7-month period; modules include an intro video, education content and suggested couple activities; emails between modules with strategies for concerns expressed during activities.
Wolff *et al*^23^	PINK! mobile app—personalised lifestyle coaching (activity tracking, psychoeducation, mindfulness) (12 weeks)	Platform: smartphone app (participants required a smartphone; the app is in German). Personalisation: described as personalised/time-independent and location-independent coaching and individualised, evidence-based content (mechanism not further specified). Coaching modules: multimodal content in nutrition, physical activity and mental health; includes therapy/side-effect management, nutritional/psychological education, mindfulness-based stress reduction and motivational exercises. Activity tracker: includes physical activity tracking; daily goals can include step counts and physical exercises. Content formats/goals: content delivered as articles, videos, and podcasts, plus daily goals (e.g., step counts, nutritional habits, exercises, and MBSR exercises). Use instructions: Patients are instructed to use the app as often as needed; there is no predefined usage time target. Data collection: questionnaires sent at 4, 8, and 12 weeks using REDCap. Usage measurement: App usage measured as minutes per week (usage groups defined over 12 weeks).

AR, augmented reality; BCSMS, Breast Cancer Self-Management Support; CBT, cognitive–behavioural therapy; CLL, Chronic Lymphocytic Leukemia; D-SCAN, Digital Supportive Care Awareness and Navigation; EHR, Electronic Health Records; ePATH, electronic Patient Activation in Treatment at Home; ePRO, electronic Patient-Reported Outcomes; ESAS-r, Edmonton Symptom Assessment System Revised; ESRA-C, ESRA-C=Electronic Self-Report Assessment for Cancer; FAQ, Frequently Asked Questions; GP, General Practitioner; HADS, Hospital Anxiety and Depression Scale; iCAN-Do, name of the internet-based stepped-care intervention evaluated in the AdultCan trial (not a true acronym); iCBT, internet-based Cognitive Behavioral Therapy; iOS, iPhone Operating System (Apple mobile operating system); MBSR, Mindfulness-Based Stress Reduction; mHealth, mobile health; MM, Multiple Myeloma; MTM, Multi-Theoretical Model of health behavior change; 6MWT, Six-Minute Walk Test; PINK!, name of the lifestyle-coaching app (not an acronym); Q&A, Questions and Answers; QoL, quality of life; REDCap, Research Electronic Data Capture; SPCT, Specialist Palliative Care Team; SxQOL, Symptom and Quality of Life; U-CARE, Uppsala University Psychosocial Care Programme; UI, user interface; UINCARE, proprietary brand name of the AR-based telerehabilitation system (not an acronym).

### Establishment and administration of telemonitoring systems in clinical practice

The way telemonitoring interventions were implemented and supervised differed across the studies. In several cases, healthcare professionals such as nurses, psychologists or dietitians played a role by offering support through virtual consultations, focusing on areas like symptom tracking, nutrition and psychological well-being.^16 26 30^ Other interventions relied on automated digital platforms or mobile apps that enabled patients to report symptoms and receive tailored responses without direct involvement from clinicians.^15 18 22^ A few studies incorporated real-time remote monitoring features, allowing healthcare providers to observe patient data through wearable devices or connected applications and respond to flagged alerts as needed.^21 27^ Additionally, some interventions included routine check-ins by the care team, either weekly or at specific intervals, to monitor patient engagement and symptom progression.^26 27 29^

### Health outcomes associated with telemonitoring

In terms of health outcomes, 6 of the 16 reviewed studies focused on anxiety and psychological distress.^15 22 23 26 29 30^ Pain management was a key outcome in several studies,^18 19 30^ and some of these reported reductions in symptom burden and improvements in QoL, although findings were not consistent across all trials. Dyspnoea, or shortness of breath, was specifically assessed in Ji *et al*,^21^ which reported enhanced breathing capacity and exercise tolerance. Sexual health and related intimacy concerns were explored in studies by Wennerberg *et al*^27^ and Wittmann *et al*.^28^ Fatigue, nausea and broader symptom distress were addressed in three studies,^18 23 30^ which reported reduced distress following the interventions. Lastly, nutritional outcomes, including weight maintenance, were the focus of Huggins *et al*,^16^ which found that telephone-based nutrition counselling was more effective than app-based support in preventing weight loss.

### Patient adherence and engagement with telemonitoring systems

Assessment methods ranged from baseline and follow-up evaluations to daily, weekly or monthly self-reports, with adherence varying across studies. Some interventions reported high engagement, such as one study where 76% of patients met feasibility criteria,^17^ while another found 73% patient satisfaction with a mobile health app.^22^ High adherence was also reported in a mobile health intervention for patients with thyroid cancer, where consistent app use over 12 weeks improved psychological outcomes.^15^ Similarly, Wolff *et al*^23^ found that higher app usage was associated with greater reductions in psychological distress and fatigue symptoms among patients with breast cancer using the PINK! app. Real-time symptom tracking, clinician feedback and automated reminders contributed to better adherence, as seen in studies using mobile applications such as ‘efil breath’ for pulmonary rehabilitation^21^ and ‘Blood Cancer Coach’ for symptom management.^22^ In contrast, interventions relying on asynchronous communication or self-guided modules showed lower engagement, as observed in a nutrition counselling study where the mobile app group had higher withdrawal rates than the telephone-based intervention.^16^ One study specifically reported excellent adherence to its ePRO-based telemonitoring protocol, with 100% of patients in the intervention arm answering ≥80% of daily app-based questions and >80% of participants maintaining this response level in each treatment week,^24^ highlighting the impact of intervention design on patient participation and sustained engagement.

### Evaluation of clinical effectiveness

To measure the clinical impact of telemonitoring interventions, studies incorporated validated outcome assessment tools. Anxiety, depression and psychological distress were evaluated using the Hospital Anxiety and Depression Scale,^29 30^ the Patient Health Questionnaire-9 (PHQ-9) in the PINK! trial,^23 26^ the PHQ-4 in the differentiated thyroid cancer trial,^15^ the Distress Thermometer^26^ and the Impact of Event Scale.^26^ Trauma-related symptoms and post-traumatic stress were assessed using the PCL-C^29^ and PCL-5.^22^ Pain management outcomes were measured using the Numerical Rating Scale and the Edmonton Symptom Assessment System Revised.^18 19^ Dyspnoea and respiratory function were assessed through the Modified Medical Research Council Score and the Six-Minute Walk Test.^21^ Cancer-specific QoL was measured using the EORTC QLQ-C30 and FACT-G, while sexual function was assessed through the International Index of Erectile Function and the Female Sexual Function Index.^27 28^ Additionally, self-efficacy in cancer management was examined using the Patient Activation Measure and the Self-Efficacy for Managing Chronic Disease Scale.^25^

## Discussion

This systematic review aimed to identify the characteristics of telemonitoring systems in cancer care, the tools used for their evaluation and the telemonitoring-associated patient clinical outcomes. The findings suggest that telemonitoring can facilitate real-time symptom tracking, may help improve adherence to treatment regimens and may enhance patient–clinician communication. However, effectiveness varied across interventions, with some studies showing limited improvements in specific clinical outcomes, such as sexual function and self-care adherence.

### Overall effectiveness of telemonitoring in cancer care

The results of this review are aligned with previous research highlighting the potential positive impact of telemonitoring in oncology care. Studies outside our review have reported that electronic symptom-reporting systems can improve health-related quality of life (HRQoL) and even overall survival in patients with advanced cancer.^30^ At the same time, within our review, some telemonitoring interventions were associated with improvements in HRQoL and reduced symptom burden,^20 25^ whereas others, such as Sprave *et al*,^24^ showed neutral effects on global QoL and, in some domains, higher reported site-specific symptom burden.

However, some findings in this review challenge existing literature by demonstrating limited effects of telemonitoring on specific clinical outcomes. For example, while pain management and respiratory function showed improvement, sexual function and intimacy-related concerns did not significantly improve despite telemonitoring interventions.^27 28^ These results contrast with a study by Reese *et al*,^31^ which suggested that structured digital interventions could enhance sexual well-being and intimacy among cancer survivors.

Additionally, our review found no significant improvements in post-treatment side effects or adherence to self-care interventions in studies such as Hoek *et al*^30^ and Wennerberg *et al*.^27^ For instance, Wennerberg et al.^27^ found that the electronic Patient Activation in Treatment at Home (ePATH) digital self-care programme for patients with prostate cancer undergoing radical prostatectomy did not result in significant improvements in urinary continence or sexual function compared with standard care. Similarly, Hoek *et al*^30^ found that teleconsultations for home-dwelling patients with advanced cancer did not significantly improve symptom burden or unmet palliative care needs, with intervention patients even reporting higher distress scores.

Moreover, the effectiveness of telemonitoring interventions may vary depending on the type of care provided and patient preferences. For example, in cases of upper gastrointestinal cancer, where disease-related malnutrition requires urgent intervention, a study comparing telephone-based dietetic counselling and a mobile app-based approach found no significant differences in outcomes over 12 months.^16^ Patients showed low acceptance of asynchronous nutrition counselling via mobile apps, highlighting that behavioural counselling alone may be insufficient for achieving nutritional adequacy. This further supports the need for personalised and synchronous telemonitoring interventions to improve adherence and clinical outcomes.

### Effectiveness of web-based telemonitoring platforms

A limited number of studies in this review reported the use of web-based interventions. Hauffman *et al*^29^ reported a small but statistically significant reduction in depressive symptoms in the internet-based stepped-care group compared with usual care, while anxiety and global QoL outcomes showed no consistent between-group differences. Similarly, Berry *et al*^18^ demonstrated that the ESRA-C, a fully automated web-based programme, effectively reduced symptom distress and enhanced patient engagement in symptom monitoring. Additionally, Wennerberg *et al*^27^ implemented a digital self-care intervention (ePATH) for patients with prostate cancer, which aimed to improve patient activation and self-care adherence. While some of these studies showed positive effects on psychological well-being and patient engagement, others, such as Hoek *et al*,^30^ found that teleconsultation-based interventions did not significantly improve symptom burden or palliative care needs. These mixed findings are consistent with a systematic review and meta-analysis on cancer survivors, which found telehealth interventions may be beneficial, although their effectiveness varies. Further high-quality research is needed to confirm their impact on Quality of Life (QoL) of patients with cancer.^32^

### Patient acceptance and feasibility of mHealth interventions

In the trials included in this review, telehealth applications were generally acceptable to many patients with cancer, although acceptance varied between interventions and populations. mHealth applications were often perceived as convenient because they are accessible via smartphones. Studies highlight the potential of mobile applications to improve treatment adherence, self-management and QoL. For instance, Hou *et al*^20^ found that the Breast Cancer Self-Management Support app significantly improved the QoL for women following a breast cancer diagnosis. These findings are supported by a meta-analysis of RCTs, which concluded that mHealth interventions are effective in improving the QoL, particularly among breast cancer survivors.^33^ Furthermore, a systematic review emphasised the role of mobile health applications in cancer pain self-management, enabling patients to track and manage their symptoms effectively.^34^ However, despite their potential, the effectiveness of mHealth interventions varies depending on patient engagement. Huggins *et al*^16^ found that a mobile-based nutrition counselling intervention had lower acceptance and higher withdrawal rates compared with a telephone-based approach. Similarly, Sprave *et al*^24^ observed consistently high adherence to ePRO symptom tracking, with all patients in the intervention arm meeting the predefined feasibility threshold and no decline in completed app-based questionnaires over time. These findings contrast with previous research suggesting that structured digital interventions generally enhance adherence and symptom self-management.^34^

### Factors affecting adherence to telemonitoring interventions

Adherence to telemonitoring interventions varied considerably across the studies included in this review. For example, Huggins *et al*^16^ reported that patients allocated to mobile app-based nutrition counselling had higher withdrawal rates and lower acceptance than those receiving telephone-based counselling, suggesting reduced engagement with asynchronous digital support. These findings appear less consistent with meta-analytic evidence from patients with severe chronic obstructive pulmonary disease, where telemonitoring has generally been associated with improved adherence and symptom self-management.^35^ However, as Jang *et al*^35^ focused on a different clinical population, their conclusions may not be directly transferable to oncology, where patterns of engagement and the burden of treatment differ substantially. Furthermore, as highlighted by Huggins *et al*,^16^ the way telemonitoring is structured seems crucial: ‘interventions that rely mainly on self-guided, asynchronous use may be more prone to disengagement than those that incorporate synchronous, real-time support from health professionals.’

More broadly, self-guided modules and asynchronous communication, where patients interact with digital tools independently, without real-time input from clinicians, appear to be associated with lower adherence in several trials, as patients may struggle to remain engaged without direct guidance, reminders or immediate feedback. In contrast, synchronous teleconsultations (eg, telephone-based counselling in Huggins *et al*^16^) provide more personalised support and accountability and can promote sustained participation. Sun *et al*^15^ similarly showed that a structured, app-based self-management programme combined with automated reminders and professional oversight was associated with sustained engagement and clinical benefits over a 12-week intervention period.

### Telemonitoring for patients and caregivers

While this systematic review focuses on telemedicine’s impact on patients with cancer, it is crucial to also consider how it can aid caregivers in their responsibilities. Only two trials have explored this aspect within their research protocols. In 2022, Merz *et al*^17 25^ conducted a study to evaluate the levels of satisfaction and QoL of patients with cancer and their caregivers while using mobile applications. The study found that the mobile app improved the Caregiver Oncology QoL Questionnaire by 2.2 points (p=0.41) and increased the use of supportive care resources for patients and caregivers. The app successfully facilitated symptom monitoring and increased awareness of cancer supportive care resources for both patients and caregivers. Similarly, LeBlanc *et al*^22^ examined a mobile health intervention for patients with blood cancer that included a caregiver support component, which led to increased awareness and use of supportive care resources among caregivers. These findings align with broader literature emphasising the importance of caregiver support in telemedicine.^36^

## Limitations

The search focused on the priorities of the eCAN Joint Action project and the thematic blocks are therefore aligned with the defined areas of interest assigned by the EU commission. This could narrow the search results, affecting the overall exhaustiveness within the existing literature.

The search focused only on patients undergoing active cancer treatment, excluding cancer survivors and those with secondary malignancies or metastatic disease. The findings are based on systems evaluated in RCTs and prospective studies, which may not represent all commercially available eHealth and mHealth technologies. Additionally, although the methodological quality of studies was generally acceptable, many were assessed as having a high risk of bias in at least one domain, especially in blinding of participants and personnel. This limitation is common in digital health research due to the nature of behavioural and self-managed interventions and may have affected participant responses or outcome reporting.

Moreover, the review primarily examined short-term to mid-term interventions, leaving long-term sustainability and effectiveness largely unaddressed. Future studies should include extended follow-up periods to better assess sustained engagement and clinical impact.

This review is limited to English-language studies, which may introduce publication bias. The studies were heterogeneous in terms of sample size, telemonitoring system type and intervention duration.

## Conclusion

Developing telemonitoring systems, delivered through mHealth and eHealth platforms, for adult patients with cancer, either as part of standard care or as complementary self-management support, presents both challenges and opportunities.

This systematic review highlights that while telemonitoring systems show promise, their effectiveness appears to vary according to the type of system, the degree of patient engagement and disease-specific needs. Across the included studies, real-time clinician feedback was often associated with better adherence and/or stronger clinical effects, whereas self-guided interventions tended to show lower engagement and higher dropout rates. Personalised telemonitoring approaches that adapt content and intensity to patient needs may help address engagement issues and could enhance long-term adherence, but these hypotheses require further testing in larger, rigorously designed trials.

## Supplementary material

10.1136/bmjcsurg-2026-000038 online supplemental file 1

10.1136/bmjcsurg-2026-000038 online supplemental file 2
