# Supplementary material for: Impact of telemonitoring systems in cancer care: a systematic review
Source: BMJ Connect Surg. 2026 Jul 9;8(1):e000038. doi: 10.1136/bmjcsurg-2026-000038 (PMC13537482; doi:10.1136/bmjcsurg-2026-000038)
Supplement: Supplementary file 1 [file bmjcsurg-8-1-s001.docx]

Supplementary File S1 — Full Search Strategies

The following table presents the precise, full search strategies applied to each database, including all search terms, Boolean operators, field codes, and limits. The search was conducted by two independent researchers (T.T. and MD.C.) in accordance with the Peer Review of Electronic Search Strategies (PRESS) (1) guidelines. All searches were conducted for the period June 2013 to March 2025.

| **Database** | **Date Searched** | **Records Retrieved** |
| --- | --- | --- |
| PubMed | June 2013 – March 2025 | 370 |
| CINAHL (EBSCO) | June 2013 – March 2025 | 120 |
| MEDLINE Complete | June 2013 – March 2025 | 113 |

Full Search String (applied to all three databases)

The following search string was applied using field codes as appropriate for each database platform (PubMed, EBSCO). The AB field code restricts terms to the abstract field; where not available, the equivalent field tag was used:

**Block 1 — Cancer / Oncology:**

AB (Neoplasm OR Cancer OR Tumor* OR Oncology OR Malignanc* OR "TELE ONCOLOGY»)

**AND**

**Block 2 — Telehealth / eHealth:**

AB (Telemedicine OR Telehealth OR eHealth OR Mhealth OR "Tele Care" OR "Virtual Medicine" OR "DIGITAL HEALTH»)

**AND**

**Block 3 — Remote monitoring / digital intervention:**

AB («Remote Consultation" OR Teleconsultation OR Telemonitoring OR Telerehabilitation OR "virtual rehabilitation" OR "remote rehabilitation" OR "internet-based" OR "web-based" OR "VIDEO BASED CONSULTATION" OR "HYBRID CANCER REHABILITATION" OR "mobile application*»)

**AND**

**Block 4 — Evaluation tools / wearables:**

All fields («Tool*" OR "assessment tool*" OR "assessment method" OR "assessing measurement tool*" OR questionnaire OR survey OR scale OR instrument OR "TELEMONITORING TOOL*" OR Wearable OR "wearable technology" OR "wearable devices" OR "wearable sensors" OR "sensor device»)

Limits and Filters Applied

| Date range | June 2013 to March 2025 |
| --- | --- |
| Language | English only |
| Publication type | Randomized controlled trials (RCTs), cluster RCTs, controlled clinical trials (CCTs), prospective clinical trials |
| Population | Adult patients (aged ≥18 years) undergoing active cancer treatment for new diagnosis or recurrence |
| Study design excluded | Cross-sectional, case study, case-control, retrospective comparative cohort designs, review formats, qualitative studies |
| Search validated by | PRESS (Peer Review of Electronic Search Strategies) guidelines |
| Search conducted by | T.T. and MD.C. independently, with K.M. as moderator for disagreements |

REFERENCE

1. McGowan J, Sampson M, Salzwedel DM, Cogo E, Foerster V, Lefebvre C. PRESS peer review of electronic search strategies: 2015 guideline statement. **J Clin Epidemiol** 2016;75:40–46.
