# Supplementary material for: Impact of telemonitoring systems in cancer care: a systematic review
Source: BMJ Connect Surg. 2026 Jul 9;8(1):e000038. doi: 10.1136/bmjcsurg-2026-000038 (PMC13537482; doi:10.1136/bmjcsurg-2026-000038)
Supplement: Supplementary file 2 [file bmjcsurg-8-1-s002.docx]

Supplementary File S2: Interrater Reliability per Screening Phase

|  |  |  |  |  |  |  | Yes Probability | No Probability | Random Agreement Probability | Cohen’s Kappa |
| --- | --- | --- | --- | --- | --- | --- | --- | --- | --- | --- |
| Screening Phase | Reviewer Pair | A Yes, B Yes | A Yes, B No | A No, B Yes | A No, B No | Proportionate Agreement |  |  |  |  |
| Title/Abstract | T.T. and MD.C. | 17 | 7 | 46 | 308 | 0.85979 (85.98%) | 0.01058 | 0.78042 | 0.79101 | 0.32911 (Fair) |
| Full-Text Review | T.T. and MD.C. | 14 | 1 | 0 | 2 | 0.94118 (94.12%) | 0.72664 | 0.02076 | 0.74740 | 0.76712 (Substantial) |
